# A novel satiety sensor detects circulating glucose and suppresses food consumption via insulin-producing cells in *Drosophila*

**DOI:** 10.1038/s41422-020-00449-7

**Published:** 2020-12-03

**Authors:** Wei Qi, Gaohang Wang, Liming Wang

**Affiliations:** 1 MOE Key Laboratory of Biosystems Homeostasis & Protection and Innovation Center for Cell Signaling Network, Life Sciences Institute, Zhejiang University, Hangzhou, Zhejiang 310058 China; 2 Institute of Molecular Physiology, Shenzhen Bay Laboratory, Shenzhen, China

**Keywords:** Calcium signalling, Nutrient signalling

## Abstract

Sensing satiety is a crucial survival skill for all animal species including human. Despite the discovery of numerous neuromodulators that regulate food intake in *Drosophila*, the mechanism of satiety sensing remains largely elusive. Here, we investigated how neuropeptidergic circuitry conveyed satiety state to influence flies’ food consumption. *Drosophila* tackykinin (DTK) and its receptor TAKR99D were identified in an RNAi screening as feeding suppressors. Two pairs of DTK^+^ neurons in the fly brain could be activated by elevated D-glucose in the hemolymph and imposed a suppressive effect on feeding. These DTK^+^ neurons formed a two-synapse circuitry targeting insulin-producing cells, a well-known feeding suppressor, via TAKR99D^+^ neurons, and this circuitry could be rapidly activated during food ingestion and cease feeding. Taken together, we identified a novel satiety sensor in the fly brain that could detect specific circulating nutrients and in turn modulate feeding, shedding light on the neural regulation of energy homeostasis.

## Introduction

Sensing hunger and satiety is crucial to ensure appropriate and balanced intake of energy and essential nutrients. In rodent models, several groups of hypothalamic neurons have been identified as satiety sensors, including those expressing pro-opiomelanocortin and those expressing melanocortin-4 receptor.^1^ These neurons survey the organismal satiety state via a number of pathways including nutrients in the circulation system, gastrointestinal fullness and fat depots, and suppress food consumption accordingly. Impaired satiety sensing may contribute to the global epidemic of obesity and other related metabolic diseases in the human society.^2^

Fruit flies *Drosophila melanogaster* offer a simple and tractable model to study satiety sensing since the key components of physiological and metabolic regulations are largely conserved between mammals and flies.^3–6^ The insulin-producing cells (IPCs) in the fly brain are one of the few well-studied regulators of feeding behavior and metabolism in fruit flies.^4^ IPCs may not be able to directly sense circulating glucose due to the lack of K_ATP_ channel,^3^ but can do so rather indirectly via other upstream neuronal populations including the adipokinetic hormone (AKH)-producing cells^7^ in the corpora cardiaca as well as a pair of glucose-sensing neurons in the fly brain expressing short neuropeptide F (sNPF) and corazonin (Crz).^8^ IPCs can also detect other nutrients in the hemolymph, including branched-chain amino acids in a cell-autonomous manner,^9^ and possibly fructose via Gr43a^+^ neurons expressing Crz in the brain.^10–12^ In addition, IPCs can reliably survey the level of fat depot in the fat body via a number of fat body-derived secretory cues, including leptin-like unpaired 2 (upd2),^13^ female-independent transformer (FIT),^14^ and Stunted (sun).^15^ In response to these satiety signals, IPCs can be activated and exert robust effects on flies’ feeding behavior and metabolism.^4^ Nevertheless, it remains unclear whether IPCs are the universal satiety sensor in the fly brain or merely function as a feeding modulator. It is also unclear how numerous feeding modulatory cues work in concert with IPCs for appropriate regulation of feeding behavior, including NPF, Hugin, DTK, Allatostatin A (AstA), drosulfakinin (DSK), and leukokinin (LK).^16^

In this study, we took a systematic approach to investigate the neural basis of satiety sensing in fruit flies, especially the involvement of neuropeptide signaling. We screened a collection of neuropeptide receptors in a quantitative food consumption assay and identified a neuropeptide receptor, tachykinin (DTK) receptor at 99D (TAKR99D), and its cognate ligand DTK, as potent feeding suppressors. We identified that two pairs of DTK^+^ neurons in the fly brain were activated by glucose in the hemolymph, which conveyed the satiety signal to IPCs via a small group of TAKR99D^+^ neurons in the pars intercerebralis (PI) region. This DTK–TAKR99D–IPC circuitry could be rapidly activated during food ingestion. Meanwhile, silencing and activating this neural circuitry exerted robust stimulatory and inhibitory effects on food consumption, respectively. Taken together, our present study revealed a novel satiety sensing and feeding regulation mechanism in fruit flies. Given the similarities of metabolic regulations between flies and mammals, our findings may also shed light on the architecture of satiety sensing mechanisms in mammals and how it is affected by metabolic diseases.

## Results and discussion

### A behavioral RNAi screening identified DTK–TAKR99D signaling as a potent feeding suppressor

To identify neuropeptidergic cues that indicated the internal satiety state and suppressed food consumption in turn, we carried out a neuron-specific RNAi screening in adult flies by using our previously developed MAFE (Manual Feeding) assay to examine food consumption. In the MAFE assay, a fine capillary carrying liquid food was presented to the proboscis of immobilized flies and the volume of consumed food was calculated after a single meal,^17^ offering a unique advantage over other established feeding assays (e.g., the CAFE assay^18^) that food consumption could be assayed independent of food-seeking activity.^5^ The use of non-nutritive L-glucose as the food source was to avoid the confounder of nutritive sugars to activate post-ingestive nutrient sensors and to modulate food consumption independent of satiety sensing.^19,20^

**Fig. 1:**
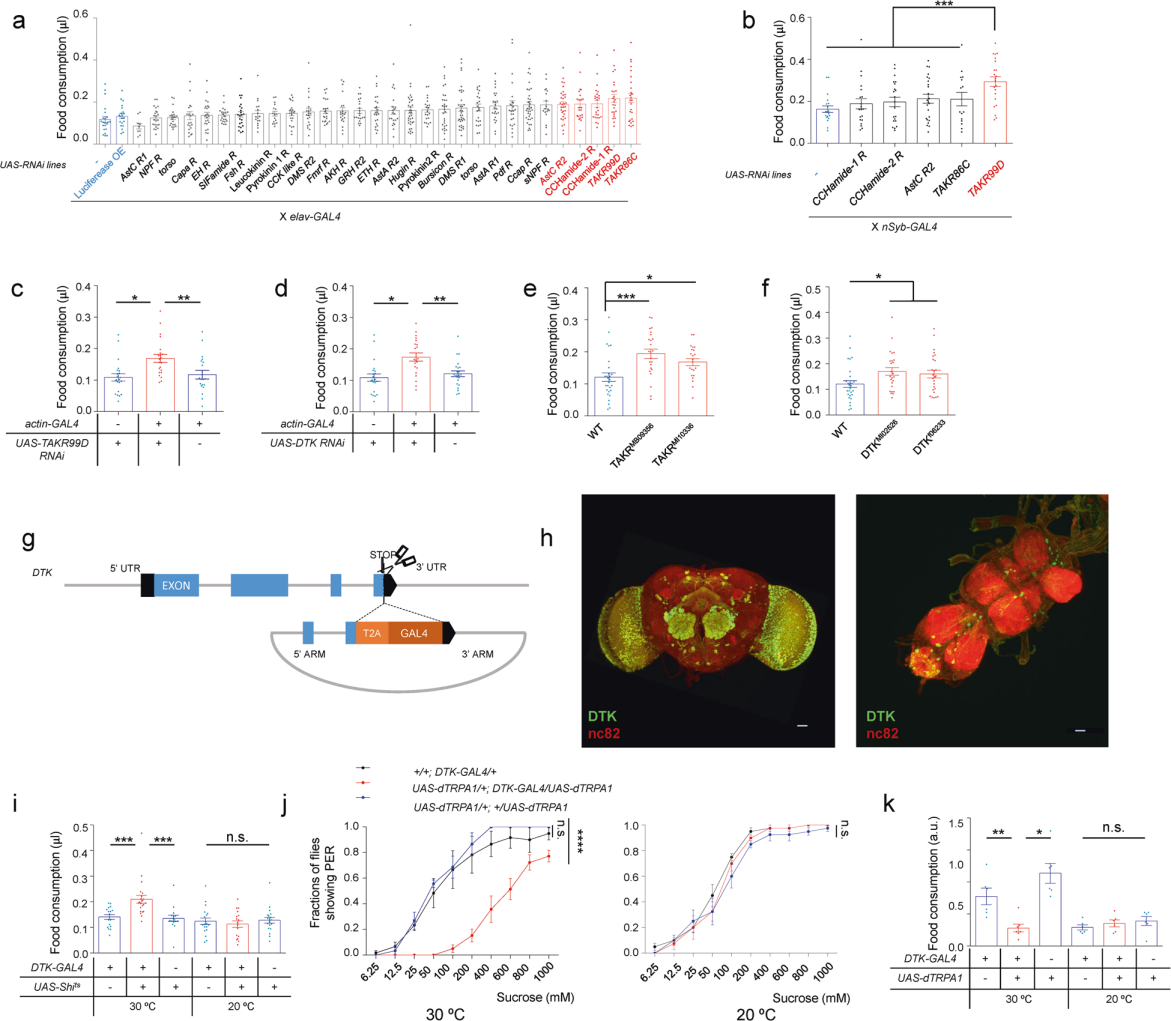
DTK–TAKR99D pathway suppresses food consumption. **a** In the pan-neuronal RNAi screening, food consumption of flies of the indicated genotypes was assayed by the MAFE assay with 500 mM L-glucose (*n* = 17–35). *elav-GAL4* was used as the pan-neuronal driver for RNAi knockdown. Empty UAS construct (−) and *UAS-luciferase* were used as controls (blue). Five different neuropeptide receptor RNAi lines showed significantly enhanced food consumption (red). **b** The volume of 500 mM L-glucose consumed by flies of the indicated genotypes. In the secondary RNAi screening, another pan-neuronal driver nSyb-GAL4 was used (*n* = 15–24). **c**–**f** The volume of 500 mM L-glucose consumed by flies of the indicated genotypes (*n* = 19–28). **g** Schematic diagram of *DTK*^*GAL4*^ allele. T2A and GAL4 coding sequences were inserted right before the stop codon of the *DTK* gene via CRISPR/Cas9-facilitated homologous recombination.^40^ **h** DTK expression in the brain (left) and the ventral nerve cord (right), illustrated by mCD8::GFP expression driven by *DTK*^*GAL4*^. Scale bars, 10 μm. **i** The volume of 500 mM L-glucose consumed by flies of the indicated genotypes at different ambient temperatures (*n* = 17–22). **j** Fractions of flies of the indicated genotypes showing PER responses to sucrose at 30 °C (left) or 20 °C (right) (*n* = 30–60). **k** Relative food consumption of flies of the indicated genotypes assayed in groups of ten flies (*n* = 6–11). Error bars represent SEM. ns, *P* > 0.05; *P < 0.05; **P < 0.01; ***P < 0.001.

Five out of 30 RNAi lines that we screened exhibited significantly increased food consumption compared to the controls (Fig. 1a, red vs blue). These positive hits were further validated in a secondary screening using a different pan-neuronal GAL4 driver, in which only the knockdown of TAKR99D was confirmed to increase food consumption (Fig. 1b, red vs blue).

TAKR99D is the cognate receptor of DTK, the fly homolog of mammalian substance P.^21^ Therefore, we hypothesized that DTK–TAKR99D signaling might be a potent feeding suppressor. Indeed, RNAi knockdown of both DTK and TAKR99D by a ubiquitous GAL4 driver, as well as the genetic mutations of these two genes, all led to an enhancement of food consumption of L-glucose (Fig. 1c–f, red vs blue). Flies bearing mutations in *Dtk* and *Takr99d* genes also exhibited enhanced food consumption of nutritive D-glucose (Supplementary information, Fig. S1a, b). These results suggest that DTK–TAKR99D signaling is required for suppressing food consumption. In contrast, disrupting DTK–TAKR99D signaling did not affect starvation-induced food-seeking behavior, or the body weight and length of fruit flies (Supplementary information, Fig. S2).

Several transgenic DTK-GAL4 lines were generated in previous studies, showing considerable variations in DTK expression patterns.^22,23^ None of these lines exhibited good colocalization with DTK antibody staining,^23^ likely indicating that they did not faithfully recapitulate endogenous DTK expression. Therefore, we generated a knock-in GAL4 allele, *DTK*^*GAL4*^, in which the GAL4 coding sequence was fused to the end of the DTK coding sequence, to better characterize DTK^+^ neurons (Fig. 1g). By using this construct, we found that DTK was widely expressed in the fly brain, heavily innervating the central complex, the antennal lobes (AL), and the optic lobes (OL) (Fig. 1h, left). There was also some expression of DTK in the ventral nerve cord (Fig. 1h, right).

Acute silencing of DTK^+^ neurons by the ectopic expression of Shibire^ts^ (Shi^ts^), a temperature-sensitive version of Dynamin,^24^ at non-permissive temperature (30 °C) led to a robust increase in food consumption of both non-nutritive L- and nutritive D-glucose, compared to the control groups (Fig. 1i and Supplementary information, Fig. S1c).

Consistently, we found that artificial activation of DTK^+^ neurons by the ectopic expression of temperature-sensitive cation channel dTRPA1^25^ resulted in reduced proboscis extension reflex (PER), the initial step of food consumption (Fig. 1j), and decreased long-term food consumption (Fig. 1k) at 30 °C. Taken together, these data suggest that DTK–TAKR99D signaling is a potent feeding suppressor.

### Two pairs of DTK^+^ neurons could be activated by glucose

The fact that DTK–TAKR99D signaling imposed a robust suppressive effect on food consumption raised the possibility that DTK^+^ neurons might be a satiety sensor and modulate feeding behavior in sated flies. Indeed, we also found that silencing DTK^+^ neurons led to increased feeding preference towards lower concentration of nutritive D-glucose over higher concentration of non-nutritive L-glucose (Supplementary information, Fig. S3), resembling the behaviors of starved flies.^26^

**Fig. 2:**
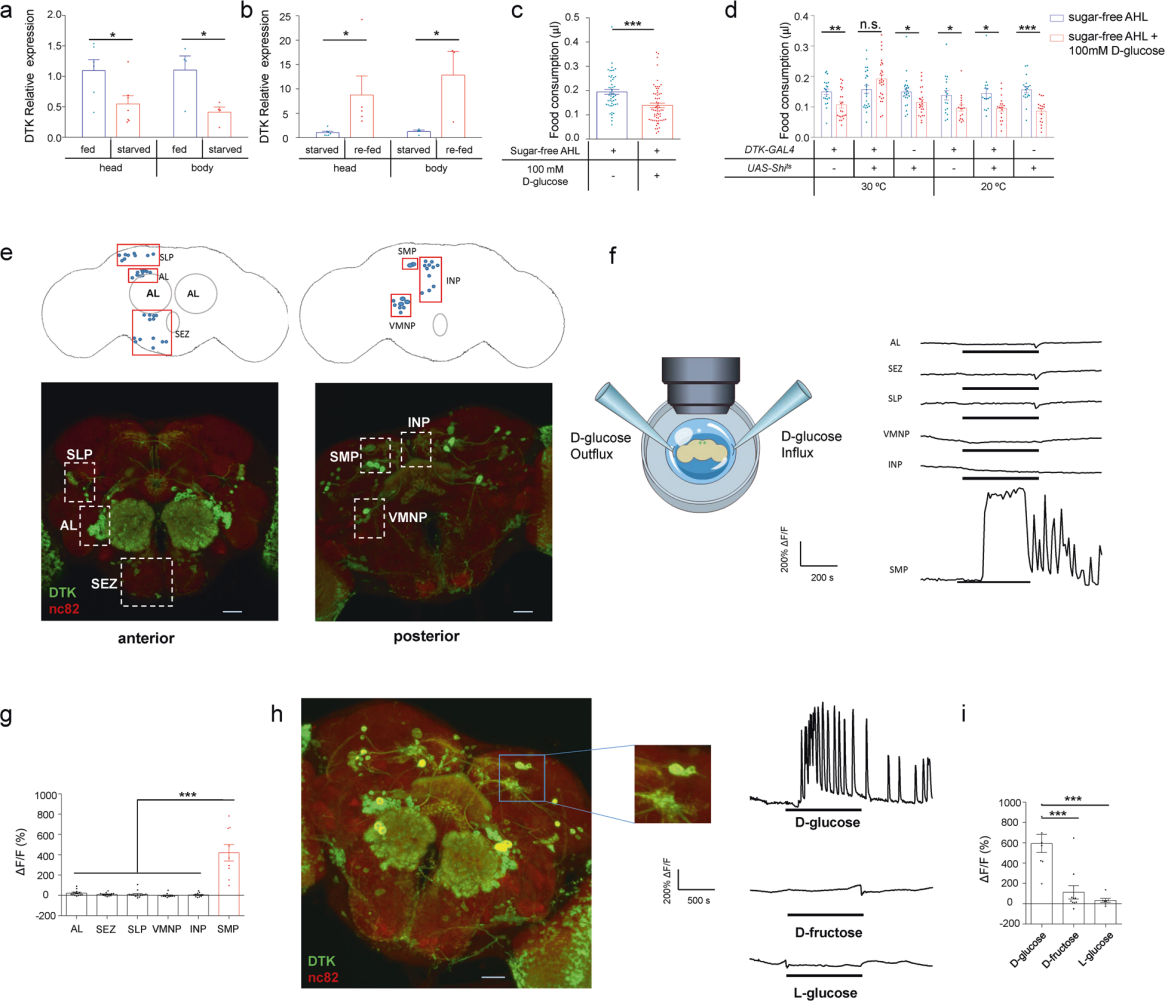
Two pairs of DTK^+^ neurons can sense glucose. **a** Relative DTK mRNA expression levels in the head and the body of flies fed ad libitum or starved for 24 h (*n* = 4–7). **b** Relative DTK mRNA expression levels in the head and the body of flies starved for 24 h with/without acute re-feeding for 1.5 h (*n* = 3–7). **c** The volume of 500 mM L-glucose consumed by starved *Canton-S* flies injected with AHL alone or AHL plus 100 mM D-glucose into the thorax (*n* = 50–63). **d** The volume of 500 mM L-glucose consumed by flies of the indicated genotypes injected with AHL alone or AHL plus 100 mM D-glucose into the thorax (*n* = 17–28). **e** DTK expression can be categorized into six clusters (boxes) in the anterior (left) and the posterior (right) parts of the fly central brain. Scale bars, 10 μm. **f** Schematic diagram of the ex vivo calcium imaging preparations (left). Representative traces of the calcium responses in each of the six neuronal clusters during the perfusion of 80 mM D-glucose (right). **g** Quantification of the calcium responses to 80 mM D-glucose from different clusters of DTK^+^ neurons (*n* = 9–14). **h** An enlarged image of DTK^+^ neurons in the SMP cluster (left). Representative traces of calcium responses to 80 mM D-glucose, D-fructose, and L-glucose from individual SMP DTK^+^ neurons shown on left (right). Horizontal black bars represent stimulus perfusion. **i** Quantification of the calcium responses to 80 mM D-glucose, D-fructose, and L-glucose from individual SMP DTK^+^ neurons. Error bars represent SEM. ns, *P* > 0.05; *P < 0.05; **P < 0.01; ***P < 0.001.

To further test this hypothesis, we quantified DTK mRNA in starved and fed flies by using quantitative RT-PCR. In both head and body tissues, DTK expression was significantly reduced by starvation (Fig. 2a). We also found that re-feeding starved flies could rapidly induce DTK expression (Fig. 2b). Therefore, DTK–TAKR99D signaling might indeed be a satiety sensor.

We then hypothesized that D-glucose, one of the major circulating sugars in flies’ hemolymph,^3^ could be the satiety cue that enhanced DTK expression and activated DTK^+^ neurons. We injected sugar-free Adult Hemolymph Like (AHL) buffer added with 100 mM D-glucose into flies’ thorax. As a result, these flies exhibited significantly decreased food consumption after the injection, compared to those injected with AHL alone (Fig. 2c). Silencing DTK^+^ neurons eliminated the effect of D-glucose injection to suppress food consumption (Fig. 2d). These data indicate that increased hemolymph D-glucose is a robust satiety signal to suppress feeding and this effect requires the activation of DTK^+^ neurons.

We next asked which specific DTK^+^ neurons were involved in the detection of D-glucose. DTK^+^ neurons in the central brain could be divided into six clusters based on the locations of their cell bodies, including AL, subesophageal zone (SEZ), superior lateral protocerebrum (SLP), superior medial protocerebrum (SMP), ventromedial neuropils (VMNP), and inferior neuropils (INP) (Fig. 2e). We performed calcium imaging in ex vivo brain preparations^19,27^ and examined calcium responses in each of these clusters upon the perfusion of D-glucose (Fig. 2f). Only the SMP cluster exhibited robust calcium responses to D-glucose (Fig. 2g). Within the SMP cluster, we then performed a more careful calcium imaging at the single-cell resolution and found that only a pair of DTK^+^ neurons of each brain hemisphere were activated by D-glucose with substantial calcium oscillations (Fig. 2h and Supplementary information, Fig. S4). The same SMP DTK^+^ neurons could not be activated by D-fructose, a minor group of circulating sugars in flies’ hemolymph,^10^ or non-nutritive L-glucose (Fig. 2h, i), highlighting the specificity of SMP DTK^+^ neurons as an internal glucose sensor. Taken together, we found two pairs of DTK^+^ neurons in the SMP cluster as a novel satiety sensor, being activated by circulating D-glucose and suppressing food consumption accordingly.

### PI TAKR99D^+^ neurons and IPCs were downstream of SMP DTK^+^ neurons

**Fig. 3:**
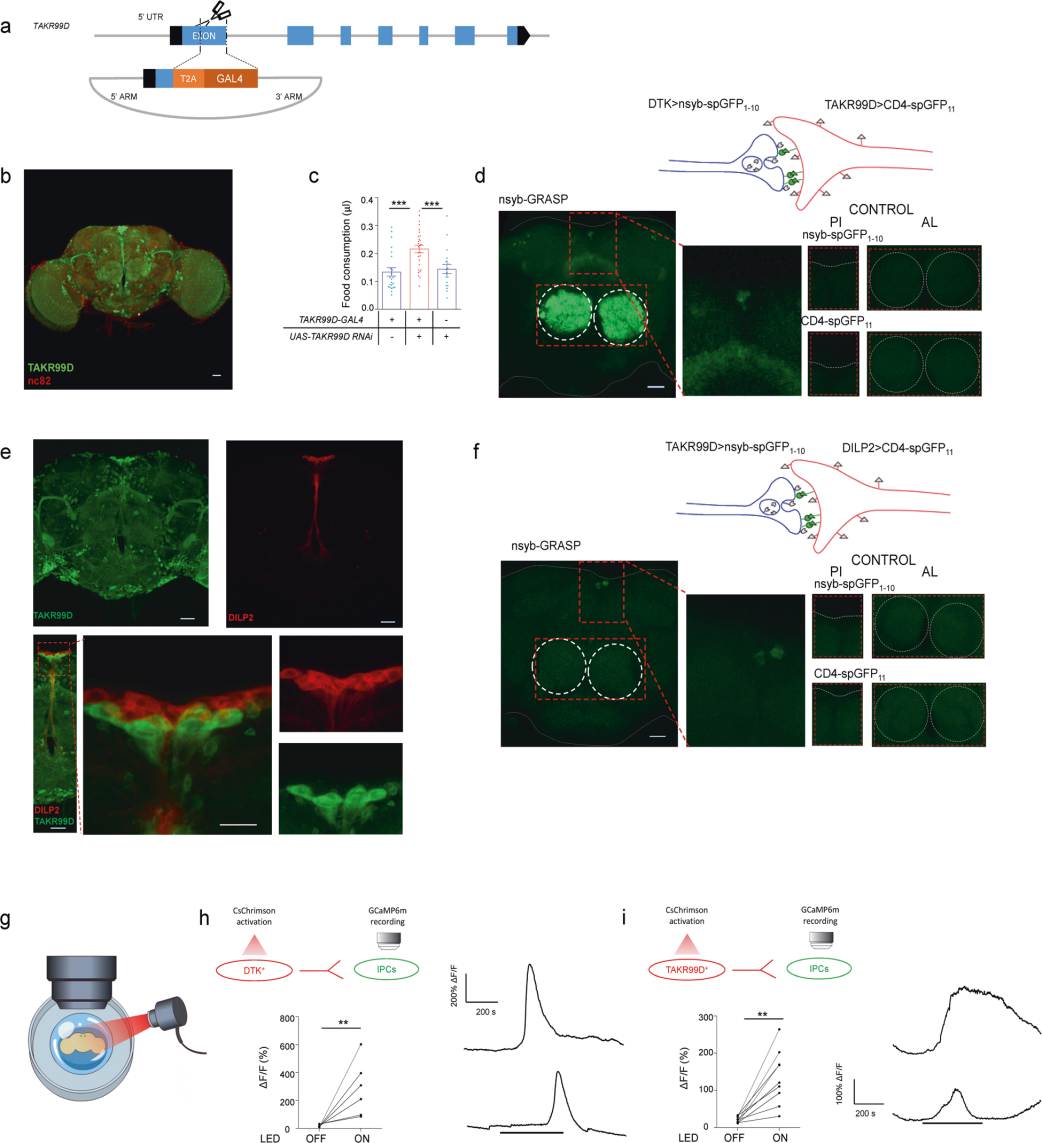
Characterization of a two-synapse neural circuitry from SMP DTK^+^ neurons to PI TAKR99D^+^ neurons to IPCs. **a** Schematic diagram of *TAKR99D*^*GAL4*^ allele. Part of the first exon of *TAKR99D* gene was replaced by T2A and GAL4 coding sequences via CRISPR/Cas9-facilitated homologous recombination. **b** TAKR99D expression in the brain, illustrated by mCD8::GFP driven by *TAKR99D*^*GAL4*^. Scale bar, 10 μm. **c** The volume of 500 mM L-glucose consumed by flies of the indicated genotypes (*n* = 19–28). **d** Schematic diagram of nSyb-GRASP between DTK^+^ and TAKR99D^+^ neurons (top). nSyb-GRASP signals between DTK^+^ neurons and TAKR99D^+^ neurons in the PI region and the AL region (left, red boxes). In the control groups lacking either of the nSyb-GRASP component, no nSyb-GRASP signal was seen (right). Scale bar, 10 μm. **e** Localization of TAKR99D^+^ neurons (green, left) and IPCs (red, right) in the brain (upper). Non-overlapping locations of TAKR99D^+^ neurons (green) and IPCs (red) in the PI region (lower). Scale bars, 10 μm. **f** Schematic diagram of nSyb-GRASP between TAKR99D^+^ and IPCs (top). nSyb-GRASP signals between TAKR99D^+^ neurons and IPCs in the PI region (left, red boxes). In the control groups lacking either of the nSyb-GRASP component, no nSyb-GRASP signal was seen (right). Scale bar, 10 μm. **g** Schematic diagram of the ex vivo optogenetics and calcium imaging preparations. **h** Quantification of the calcium responses of IPCs upon the activation of DTK^+^ neurons (*n* = 6) (left). Representative traces of the calcium responses of IPCs (right). Horizontal black bar represents the duration of red light stimulation. **i** Quantification of the calcium responses of IPCs upon the activation of TAKR99D^+^ neurons (*n* = 8) (left). Representative traces of the calcium responses of IPCs (right). Horizontal black bar represents the duration of red light stimulation. Error bars represent SEM. **P < 0.01; ***P < 0.001.

Next, we sought to examine the downstream targets of DTK^+^ neurons to suppress food consumption. We had already identified one DTK receptor, TAKR99D, as a potent feeding suppressor (Fig. 1). Therefore, TAKR99D^+^ neurons could be the direct downstream target of DTK^+^ neurons. We generated a *TAKR99D*^*GAL4*^ allele in which part of the first exon of *Takr99d* gene was replaced by the GAL4 coding sequence (Fig. 3a). Expression analysis using mCD8::GFP reporter revealed that TAKR99D also had a widespread distribution in the fly brain (Fig. 3b). RNAi knockdown of TAKR99D in TAKR99D^+^ neurons enhanced food consumption (Fig. 3c), further confirming the validity of this *TAKR99D*^*GAL4*^ allele.

We next asked whether DTK^+^ neurons had direct synaptic innervations with TAKR99D^+^ neurons. We used nSyb-GRASP to examine direct synaptic innervations between these two groups of neurons (Fig. 3d, top), in which neuronal synaptobrevin (nSyb) tagged with spGFP1–10 (nSyb::spGFP1–10) was expressed in DTK^+^ neurons and CD4-spGFP11 was expressed in TAKR99D^+^ neurons.^28^ Reconstituted GFP signals were detected in several brain regions including the AL, the PI, and the fan-shaped body (FB) (Fig. 3d, bottom left, dotted red boxes), but not in the control flies carrying either of the two nSyb-GRASP elements (Fig. 3d, bottom right). As reported previously, the AL innervations were likely to mediate the effect of starvation on olfaction-guided food search.^29^ We hypothesized that PI TAKR99D^+^ neurons might be the direct downstream target of SMP DTK^+^ neurons since neurons with neuroendocrinal functions are enriched in that region.^16^ Indeed, SMP DTK^+^ neurons sent their neurites to the PI region as revealed by photoactivatable (PA)-GFP expression (Supplementary information, Fig. S5).^30^ Both DTK^+^ neurons and TAKR99D^+^ neurons also projected to the FB region (Figs. 1h and 3b), but the function of their FB innervations (Fig. 3d) remained unclear and would be of interest for future studies.^31^

There were six TAKR99D^+^ neurons located in the PI region, in close proximity to IPCs (Fig. 3e, upper). However, we did not see any overlap between these two neuronal populations (Fig. 3e, lower). We therefore asked whether IPCs could be the downstream target of PI TAKR99D^+^ neurons. Indeed, nSyb-GRASP analysis confirmed that TAKR99D^+^ neurons and IPCs had direct synaptic innervations in the PI region (Fig. 3f). In contrast, although a previous report suggesting that IPCs also express DTK receptors,^32^ DTK^+^ neurons and IPCs did not have direct synaptic connections as shown by the nSyb-GRASP assay (Supplementary information, Fig. S6).

We then asked whether this DTK–TAKR99D–IPC circuitry had active synaptic transmissions. We ectopically expressed CsChrimson, a red-shifted channelrhodopsin variant,^33^ in either DTK^+^ neurons or TAKR99D^+^ neurons, and GCaMP6m in IPCs. Photoactivation of DTK^+^ neurons elicited robust calcium responses in IPCs (Fig. 3g, h; Supplementary information, Fig. S7a). Similar effect was observed when activating TAKR99D^+^ neurons and imaging the calcium responses in IPCs (Fig. 3i and Supplementary information, Fig. S7b). In contrast, in the absence of CsChrimson, red light stimulation of either DTK^+^ or TAKR99D^+^ neurons did not elicit calcium responses in IPCs (Supplementary information, Fig. S8). Collectively, these data uncover a two-synapse neural circuitry formed by SMP DTK^+^ neurons, PI TAKR99D^+^ neurons, and IPCs, which may function as a satiety sensor to detect circulating D-glucose and to inhibit food consumption.

### TAKR99D^+^ neurons and IPCs were activated by dietary nutrients to cease food consumption

We next sought to examine the physiological significance of this DTK–TAKR99D–IPC circuitry in regulating flies’ food consumption. Like SMP DTK^+^ neurons, PI TAKR99D^+^ neurons as well as IPCs could be activated by D-glucose perfusion in the ex vivo calcium imaging preparations (Supplementary information, Fig. S9). Since this DTK–TAKR99D–IPC circuitry could be activated by D-glucose in the ex vivo brain preparations, in the absence of peripheral sensory organs, we reasoned that it might function as an internal glucose sensor. More specifically, we hypothesized that during the actual feeding episodes this neural circuitry could be activated by ingested nutrients and rapidly impose a suppressive effect on food consumption, ensuring the appropriate amount of food being ingested.

**Fig. 4:**
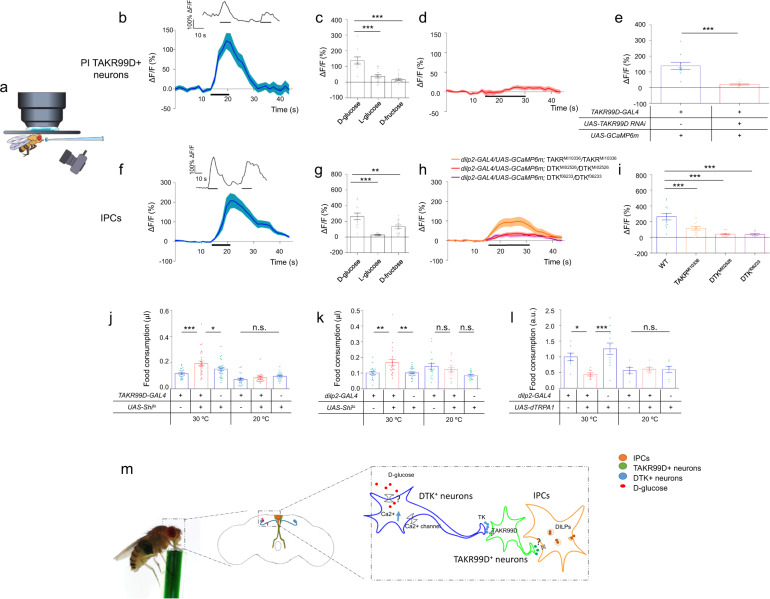
DTK–TAKR99D–IPC circuitry is a satiety sensor and a feeding suppressor. **a** Schematic diagram of the in vivo calcium imaging preparations. **b**, **f** Averaged (bottom) and representative (top) traces of the calcium responses of PI TAKR99D^+^ neurons (**b**) and IPCs (**f**) upon the ingestion of nutritive D-glucose (*n* = 10–12). Horizontal black bars represent the feeding episodes. **c, g** Quantification of the calcium responses of PI TAKR99D^+^ neurons (**c**) and IPCs (**g**) upon the ingestion of D-glucose, L-glucose, and D-fructose (*n* = 10–16). **d**, **h** The averaged calcium responses of PI TAKR99D^+^ neurons (**d**) and IPCs (**h**) of the indicated genotypes upon the ingestion of D-glucose (*n* = 10–16). Horizontal black bars represent the feeding episodes. **e**, **i** Quantification of the calcium responses of PI TAKR99D^+^ neurons (**e**) and IPCs (**i**) of the indicated genotypes upon the ingestion of D-glucose (*n* = 10–16). **j**, **k** The volume of 500 mM L-glucose consumed by flies of the indicated genotypes (*n* = 20–31). **l** Relative food consumption of flies of the indicated genotypes assayed in groups of 10 flies (*n* = 6–11). Error bars represent SEM. ns, *P* > 0.05; *P < 0.05; **P < 0.01; ***P < 0.001. **m** A working model. During the ingestion of nutritive sugars (e.g., D-glucose, red), two pairs of SMP DTK^+^ neurons (blue) are activated due to elevated circulating glucose in the hemolymph. As a result, DTK is released from these neurons to activate downstream TAKR99D^+^ neurons in the PI region (green), which subsequently elicits calcium responses in IPCs (orange). IPCs, as the satiety center, cease food intake behaviors via its downstream neural circuitry.

To test this hypothesis, we first performed in vivo calcium imaging of PI TAKR99D^+^ neurons and IPCs in feeding flies (Fig. 4a). The ingestion of nutritive sugar D-glucose could strongly activate PI TAKR99D^+^ neurons (Fig. 4b and Supplementary information, Fig. S10a) and IPCs (Fig. 4f and Supplementary information, Fig. S10b). It is likely that during these feeding episodes, dietary D-glucose was quickly absorbed and transported into the hemolymph and activated DTK–TAKR99D–IPC circuitry.

Similar to SMP DTK^+^ neurons, PI TAKR99D^+^ neurons and IPCs exhibited significantly weaker calcium responses to D-fructose and L-glucose than D-glucose (Fig. 4c, g), suggesting that they are specific D-glucose sensors. Detailed analysis of the neuronal calcium responses before vs during the feeding episodes further showed that TAKR99D^+^ neurons were responsive to D-glucose feeding but not to D-fructose or L-glucose feeding (Supplementary information, Fig. S11a–c). IPCs exhibited strong calcium responses to D-glucose feeding in the same experiments (Supplementary information, Fig. S11d). Unlike TAKR99D^+^ neurons, IPCs were activated during fructose feeding (Supplementary information, Fig. S11f), which was likely mediated by pre-synaptic input from Gr43a^+^ neurons in the brain.^11,12^ Gr43a^+^ brain neurons were reported to be fructose sensitive and to innervate with IPCs.^10–12^ We also found that IPCs exhibited a very weak but nevertheless statistically significant response to L-glucose (Supplementary information, Fig. S11e). The underlying mechanism remained unclear, though the L-glucose responses of IPCs were unlikely to be mediated by TAKR99D^+^ neurons (Supplementary information, Fig. S11b, e).

Therefore, DTK–TAKR99D–IPC circuitry could rapidly sense the dietary intake of D-glucose and hence the satiety state, in the time scale of seconds. Previous work from our lab and others have shown that a typical meal of fruit flies often lasted only 10–20 s,^17,34^ and a single feeding episode lasted only 1–2 s.^35^ Therefore, a satiety sensor that works in the time scale of seconds is needed for appropriate feeding control of fruit flies. It is worth noting that for both PI TAKR99D^+^ neurons and IPCs, the calcium responses to D-glucose were much slower in the ex vivo brain preparations (Supplementary information, Fig. S9) than in the in vivo setup (Fig. 4b, f). This seems to be a common problem for ex vivo calcium imaging experiments in the fly brain^8,19,20^ and we speculated that this might be due to differences in the kinetics of stimulus penetration.

We next asked whether the activation of PI TAKR99D^+^ neurons and IPCs by dietary D-glucose required DTK–TAKR99D signaling. RNAi knockdown of TAKR99D in TAKR99D^+^ neurons completely abolished their responses to D-glucose (Fig. 4d, e). Similarly, in flies bearing mutations in *Dtk* and *Takr99d* genes, IPCs exhibited much reduced calcium responses to D-glucose (Fig. 4h, i). These results suggest that DTK–TAKR99D signaling is required for the DTK–TAKR99D–IPC circuitry to function as a satiety sensor, sensing D-glucose and in turn suppressing food consumption.

Consistent with the role of a satiety sensor, TAKR99D^+^ neurons and IPCs exerted strong suppressive effects on food consumption. Silencing both groups of neurons led to enhanced food consumption, mimicking the effect of silencing DTK^+^ neurons (Fig. 4j, k). Conversely, activating IPCs reduced food consumption (Fig. 4l), phenocopying the activation of DTK^+^ neurons. Activation of TAKR99D^+^ neurons was lethal, suggesting that some other clusters of TAKR99D^+^ neurons might be essential for survival.

In summary, our work has identified a novel satiety sensor in the fly brain that can sense the increase in circulating glucose during food ingestion and suppress further food intake. This satiety sensor is composed of three clusters of neurons that form a two-synapse neural circuitry: two pairs of DTK^+^ neurons in the SMP region, six TAKR99D^+^ neurons in the PI region, and fourteen IPCs. This circuitry can rapidly feed the information of the satiety state to IPCs and prevent flies from overfeeding, thereby contributing to the maintenance of energy homeostasis in flies (Fig. 4m). It will be of great interest to further study how this novel satiety sensor rapidly responds to the rise and fall of circulating D-glucose during a meal and modulate food consumption in a timely manner.

To accurately survey the overall satiety state of a living organism, the “satiety center” needs to sample the internal nutrient storage of different types, from different locations, and via different sensing pathways. Our present study uncovers a novel pathway of satiety sensing in fruit flies. Future studies are needed to understand how it interacts with other satiety sensors, especially other sugar-sensing pathways that also target IPCs directly or indirectly.^3,8,10,36,37^ Our study may also shed light on satiety sensing mechanisms in mammals and how they are affected by metabolic disturbances.

## Materials and methods

### Flies

Flies were grown on standard fly medium made of yeast, corn and agar at 25 °C and 60% humidity on a 12 h light/12 h dark cycle. Virgin female flies were collected shortly after eclosion and kept on food for 5–7 days till experiments. For experiments that required temperature treatments (Shi^ts^ and dTRPA1), flies were raised at 18 °C for 7–9 days and then transferred to 30 °C right before the behavioral tests.

All *UAS-RNAi* lines used in the screening (#36303, #35788, #25832, #25858, #25925, #25935, #25936, #25939, #25940, #26017, #27275, #27280, #27494, #27669, #27506, #27507, #27509, #27513, #27529, #27539, #38346, #28580, #28780, #28781, #28783, #29414, #29577, #29624, #31490, #34947, #31884, #31958, #33627, #35251, #35316, #35639, #38347), *UAS-TK RNAi* (#25800), *elav-GAL4* (#25750), *TAKR*^*MB09356*^ (#26471), *TAKR*^*MI10336*^ (#54533), *DTK*^*MI02526*^ (#35996), *DTK*^*f06233*^ (#22368), *UAS-mCD8::GFP* (#5137), *LexAop-rCD2RFP,UAS-mCD8GFP* (#67093), *UAS-GCaMP6m* (#42748), *LexAop-GCaMP6m* (#44275), *UAS-nSyb-spGFP1–10,LexAop-CD4-spGFP11* (#64314), *LexAop-nSyb-spGFP1–10,UAS-CD4-spGFP11* (#64315), and *dilp2-GAL4* (#37516) were obtained from the Bloomington *Drosophila* Stock Center at Indiana University. *UAS-dTRPA1*, *UAS-C3PA-GFP,UAS-Shi*^*ts*^, and *UAS-CsChrimson* were from David Anderson (Caltech). *dilp2-LexA* was a gift from Zhefeng Gong (Zhejiang University, China). All flies were backcrossed for at least six generations before use to ensure that the genetic backgrounds were comparable.

### The MAFE assay

As described previously,^17^ flies were starved in plastic vials containing 2% agar for 30–40 h till the assay. Individual flies were transferred and immobilized in a 200 μL pipette tip, and then sated with sterile water before being presented with 500 mM L-glucose filled in a graduated glass capillary (VWR, #53432-604). The food stimulation was repeated until the flies became unresponsive to a series of ten food stimuli, and the total food consumption was calculated afterwards.

### The PER assay

The PER assay was conducted as described previously.^17^ Flies were prepared as in the MAFE assay and subjected to different sugar solutions, twice for each concentration. Flies showing PER responses to at least one of the two trials were considered positive to that sugar concentration.

### Dye-labeled feeding assay

As described previously,^38^ flies (in groups of ten) were starved for 30 h on 2% agar before being transferred to vials containing 500 mM L-glucose (or 500 mM D-glucose if indicated), 2% agar, and 1% (w/v) FD&C Blue #1. After 1 h, flies were homogenized and the absorbance of the supernatant was measured at 630 nm (A630).

### Locomotion assay

As described in our previous work,^39^ the locomotor activity of individual flies was monitored using DAMS (Trikinetics). Briefly, individual flies were anesthetized and introduced into a 5 mm × 65 mm polycarbonate tube (Trikinetics); one end of the tubes was filled with 2% agar or 2% agar + 5% sucrose and the other end was blocked by cotton wool. These tubes were inserted into DAMS monitors and kept in incubators during experiments. The frequency of these flies to walk across the infrared beam placed in the center of the tubes was collected as an indirect measure of their locomotion.

### AHL injection

Flies were immobilized as previously described.^17^ Approximately 50 nL of AHL buffer (108 mM NaCl, 8.2 mM MgCl_2_, 4 mM NaHCO_3_, 1 mM NaH_2_PO_4_, 2 mM CaCl_2_, 5 mM KCl, 5 mM HEPES)^20^ with or without the addition of 100 mM D-glucose was injected into the thorax of these flies with a glass micropipette. The glass micropipette was pulled from thick-walled borosilicate capillaries (Sutter Instruments, BF120-69-10) with a micromanipulator (Sutter Instruments, MP225) to control its movements. Flies’ food consumption was measured immediately after AHL injection by the MAFE assay.

### Two-choice feeding assay

Two-choice behavioral assay was conducted as previously described.^20^ Approximately 25 flies fed *ad libitum* were placed into a 60-well mini tray (Nunc, #439225). The substrate in each well was made of 1% agar containing a sugar as indicated and labeled with different food dyes. The flies were allowed to feed for 30 min at room temperature in dark. The color of the abdomen of the flies was assessed and the preference index (% PI) was determined using the following equation: % PI = [(No. of flies ate sugar 1 + 0.5 × No. of flies ate both) – (No. of flies ate sugar 2 + 0.5 × No. of flies ate both)]/(No. of flies ate either or both sugars).

### Calcium imaging

For the ex vivo calcium imaging preparations, adult fly brains were dissected in the sugar-free AHL buffer and mounted on Poly-L-Lysine (PLL; Sigma Aldrich, P1524) coated cover glass. For experiments shown in Fig. 2f, each frame contained ten scans which recorded the whole fly brain from anterior to posterior and each frame took ~15 s. The first 20 frames were recorded when perfusing sugar-free AHL as the baseline, the next 40 frames were recorded during D-glucose perfusion, and the last 40 frames were recorded during AHL washout. For experiments shown in Fig. 2h, the dissected fly brain was bathed in the sugar-free AHL and recorded for 100 frames (5 s per frame) to establish a baseline. Afterwards, the next 200 frames were recorded during D-glucose perfusion and the following 200 frames were recorded during AHL washout.

For in vivo calcium imaging preparations,^19^ adult flies were anesthetized on ice and then glued onto a transparent tape. The cuticle of the dorsal part of the fly head was gently removed with forceps and the exposed brain was bathed in the sugar-free AHL. Liquid food was delivered to the proboscis of flies by a micromanipulator (Sutter Instruments, MP225). The flies were illuminated by an IR LED (Thorlabs, M850L3) and the feeding behavior was imaged by a CMOS camera (FLIR USB 2.0, Point Gray) with an amplify lens (NAVITAR, 1-60135-IR). The calcium signals were recorded at 1 frame/s.

For all calcium imaging experiments, the calcium signals were recorded by a Nikon C2 confocal microscope with a water immersion objective lens (40× /0.80w DIC N2). Image analyses were performed in ImageJ and plotted in Graphpad Prism 6 or Matlab (MathWorks). The florescent signal changes were calculated using the following formula: ΔF/F = [F – F_0_]/F_0_, where F was the mean florescence of cell body and F_0_ was the baseline.

### Immunohistochemistry

Immunohistochemistry was conducted according to the protocol from Janelia Farm Research Campus (https://www.janelia.org/project-team/flylight). Briefly, flies were dissected in Schneider’s Insect Medium (Sigma Aldrich, S0146) and fixed with 2% PFA in S2 medium for 55 min. The fixed samples were washed with Washing Buffer (0.5% Triton X-100 in PBS) for 4 × 15 min, incubated with Penetration/Blocking Buffer (5% Calf Serum and 0.5% Triton X-100 in PBS) for 1.5 h at room temperature, and then incubated with primary antibodies for 36–48 h at 4 °C. The samples were washed for 4 × 15 min and incubated with secondary antibodies for 72 h at 4 °C. The brains were again fixed with 4% PFA and mounted on PLL-coated cover glass. This step was followed by ethanol dehydration and xylene clearing, and DPX was then added to the brain side of the cover glass. The samples were finally mounted on a microscope slide.

Samples were imaged with Nikon 20× /0.45 and 40× /0.80w. Antibodies were used at the following dilutions: mouse anti-nc82 (1:400, DSHB), rabbit anti-GFP (1:800, Life Technologies), rabbit anti-DsRed (1:1000, ClonTech), mouse anti-GFP (1:1000, Abcam), Alexa Fluor 546 goat anti-mouse (1:1000, Life Technologies), Alexa Fluor 488 goat anti-rabbit (1:1000, Life Technologies), Alexa Fluor 488 goat anti-mouse (1:1000, Life Technologies), Alexa Fluor 546 goat anti-rabbit (1:1000, Life Technologies).

### nSyb-GRASP

To examine nSyb-GRASP signals, the fly brains were dissected in PBS and fixed in 4% PFA for 55 min at room temperature. After fixation, the samples were washed, mounted and dehydrated as described in the immunohistochemistry experiment. Images were acquired using Nikon 40× /0.80w water immersion objective at 512 × 512 pixel resolution and 1 μm intervals.

### PA-GFP

PA-GFP experiments were performed with a two-photon laser-scanning microscope (Olympus). *+/+; TK-GAL4/UAS-C3PA-GFP, UAS-mKO2* flies were dissected in AHL and the brains were mounted on PLL-coated cover glass, which was then immersed in AHL buffer. The images of the pre-photoactivated brains were obtained at 920 nm. The cell bodies of SMP DTK^+^ neurons as illustrated by mKO2 signal were photoactivated by two cycles of exposure (10 scans × 60 repeats per cycle) to 720 nm laser. There was a 10 min interval after the first photoactivation period. Afterwards, these samples were incubated for 20 min after two photoactivation cycles. Images were then obtained with 512 × 512 resolution. Three-dimensional images were reconstructed using Fiji software.

### Quantitative RT-PCR

Flies that were fed ad libitum, starved for 24 h, and starved for 24 h followed by 2 h re-feeding were dissected to separate the heads from the bodies. The obtained heads and bodies were homogenized and the total RNA was purified with TRIzol Reagent (Life Technologies). The eluted RNA products were then reverse-transcribed with TransScript cDNA Synthesis SuperMix (TransGen). The quantitative PCR experiments were performed using a CFX96 Real-Time System (Biorad).

Primers used are:

DTK, Forward: GCTCTCTCCGATTTCTGGCA; Reverse: GAGCTTGCTCATGATCGTCAC

Actin, Forward: GAGGCTTGCGGCATCCACGAGACCAC; Reverse: GACAGAGTACTTGCGCTCTGGCG

### Optogenetics

Newly enclosed virgin female flies were collected in a fresh vial with standard medium for 3 days and then transferred into a vial with regular food containing 400 μM all-trans-retinal (Sigma, R2500) for 2–4 days till experiments. To activate the neurons expressing CsChrimson, flies were dissected and mounted as described in ex vivo calcium imaging experiments. An array of red LED (Thorlabs, M625L3) was placed above the brain, calcium signals were recorded for 100 frames (2 s per frame) without light to establish a baseline, the next 200 frames were recorded during opto-activation with light switched on and the following 200 frames were recorded without light afterwards.

### Statistical analysis

Data presented in this study were all verified for normal distribution by D’Agostino-Pearson omnibus test. Student’s *t*-test (for pairwise comparisons) and one-way ANOVA (for comparisons among three or more groups) were used. The post hoc test with Bonferroni correction was performed for multiple comparisons following ANOVA. For the RNAi screening (Fig. 1a), one-way ANOVA followed by post hoc Dunnett’s test was applied to identify hits (red) that were significantly different from the control lines (blue).

## Supplementary information

Supplementary information, Figure S1

Supplementary information, Figure S2

Supplementary information, Figure S3

Supplementary information, Figure S4

Supplementary information, Figure S5

Supplementary information, Figure S6

Supplementary information, Figure S7

Supplementary information, Figure S8

Supplementary information, Figure S9

Supplementary information, Figure S10

Supplementary information, Figure S11
