# Supplementary material for: A novel satiety sensor detects circulating glucose and suppresses food consumption via insulin-producing cells in Drosophila
Source: Cell Res. 2020 Dec 3;31(5):580–8. doi: 10.1038/s41422-020-00449-7 (PMC8089096; doi:10.1038/s41422-020-00449-7)
Supplement: Supplementary file 1 — Supplementary information, Figure S1 [file 41422_2020_449_MOESM1_ESM.pdf]

Fig S1

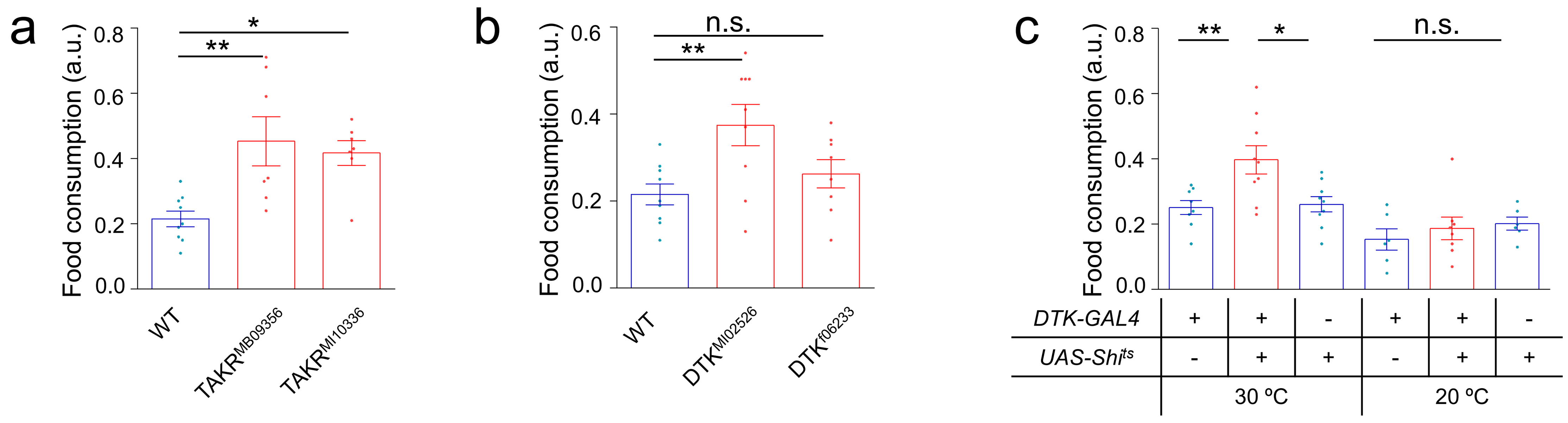
a
Food consumption (a.u.)
WT
TAKR^MB09356
TAKR^MI10336
b
n.s.
WT
DTK^MI02526
DTK^f06233
c
n.s.
DTK-GAL4
UAS-Shi^ts
30 ºC
20 ºC

Fig. S1 DTK-TAKR99D signaling suppresses D-glucose consumption. a-c Relative D-glucose consumption of flies of the indicated genotypes assayed in groups of 10 flies (n = 7–9).
