# Supplementary material for: A novel satiety sensor detects circulating glucose and suppresses food consumption via insulin-producing cells in Drosophila
Source: Cell Res. 2020 Dec 3;31(5):580–8. doi: 10.1038/s41422-020-00449-7 (PMC8089096; doi:10.1038/s41422-020-00449-7)
Supplement: Supplementary file 2 — Supplementary information, Figure S2 [file 41422_2020_449_MOESM2_ESM.pdf]

Fig S2

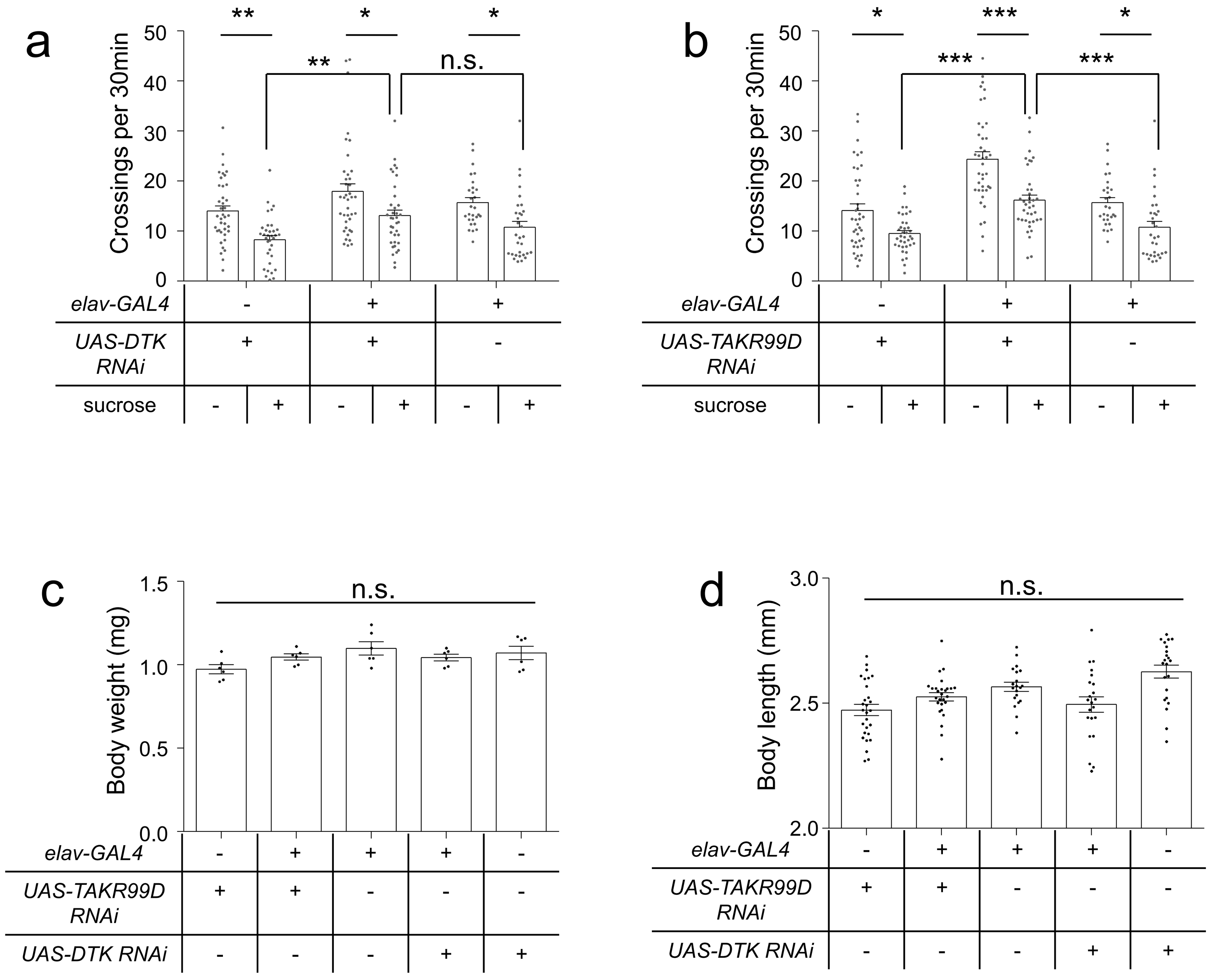

a
b
c
d
Crossings per 30min
Body weight (mg)
Body length (mm)
n.s.
elav-GAL4
UAS-DTK RNAi
UAS-TAKR99D RNAi
sucrose

Fig. S2 Disrupting DTK-TAKR99D signaling does not affect starvation-induced food-seeking behavior, body weight, or body size. a, b Average midline crossing activity of flies with pan-neuronal RNAi knockdown of DTK (**a**) or TAKR99D (**b**) (n = 31–40). **c, d** The body weight (**c**, n = 60) and the length (**d**, n = 20–28) of the indicated genotypes. Error bars represent SEM. ns, $P > 0.05$; *$P < 0.05$; **$P < 0.01$; ***$P < 0.001$.
