# Supplementary material for: A novel satiety sensor detects circulating glucose and suppresses food consumption via insulin-producing cells in Drosophila
Source: Cell Res. 2020 Dec 3;31(5):580–8. doi: 10.1038/s41422-020-00449-7 (PMC8089096; doi:10.1038/s41422-020-00449-7)
Supplement: Supplementary file 3 — Supplementary information, Figure S3 [file 41422_2020_449_MOESM3_ESM.pdf]

Fig S3

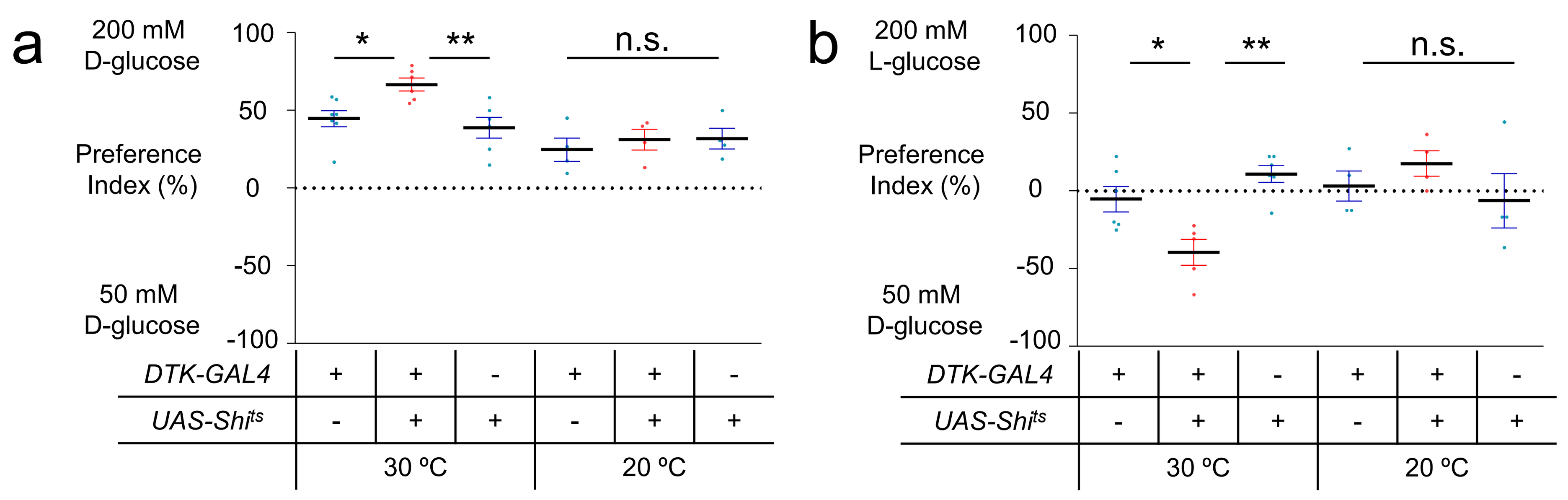

a
200 mM D-glucose
Preference Index (%)
50 mM D-glucose
100
50
0
-50
-100
*
**
n.s.
DTK-GAL4
UAS-Shi^{ts}
30 ºC
20 ºC
b
200 mM L-glucose
Preference Index (%)
50 mM D-glucose

Fig. S3 Disrupting DTK-TAKR99D signaling increases feeding preference towards nutritive sugars. a, b Feeding preference between two indicated food choices of flies of the indicated genotypes assayed in groups of 25 (n = 4–6).
