# Supplementary material for: A novel satiety sensor detects circulating glucose and suppresses food consumption via insulin-producing cells in Drosophila
Source: Cell Res. 2020 Dec 3;31(5):580–8. doi: 10.1038/s41422-020-00449-7 (PMC8089096; doi:10.1038/s41422-020-00449-7)
Supplement: Supplementary file 4 — Supplementary information, Figure S4 [file 41422_2020_449_MOESM4_ESM.pdf]

Fig S4

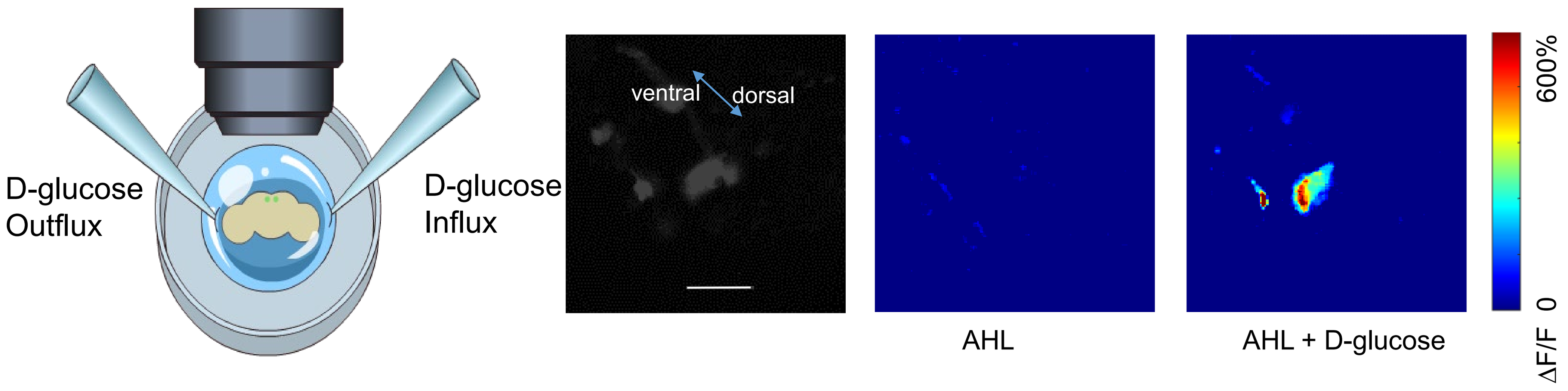

D-glucose
Outflux
D-glucose
Influx
ventral
dorsal
AHL
AHL + D-glucose
600%
0
ΔF/F

Fig. S4 DTK$^+$ neurons can be activated by D-glucose. In the *ex vivo* calcium imaging preparations (left), the perfusion of 80 mM D-glucose elicited robust calcium responses in DTK$^+$ neurons in the SMP cluster. Scale bar, 10 μm.
