# Supplementary material for: A novel satiety sensor detects circulating glucose and suppresses food consumption via insulin-producing cells in Drosophila
Source: Cell Res. 2020 Dec 3;31(5):580–8. doi: 10.1038/s41422-020-00449-7 (PMC8089096; doi:10.1038/s41422-020-00449-7)
Supplement: Supplementary file 5 — Supplementary information, Figure S5 [file 41422_2020_449_MOESM5_ESM.pdf]

Fig S5

posterior

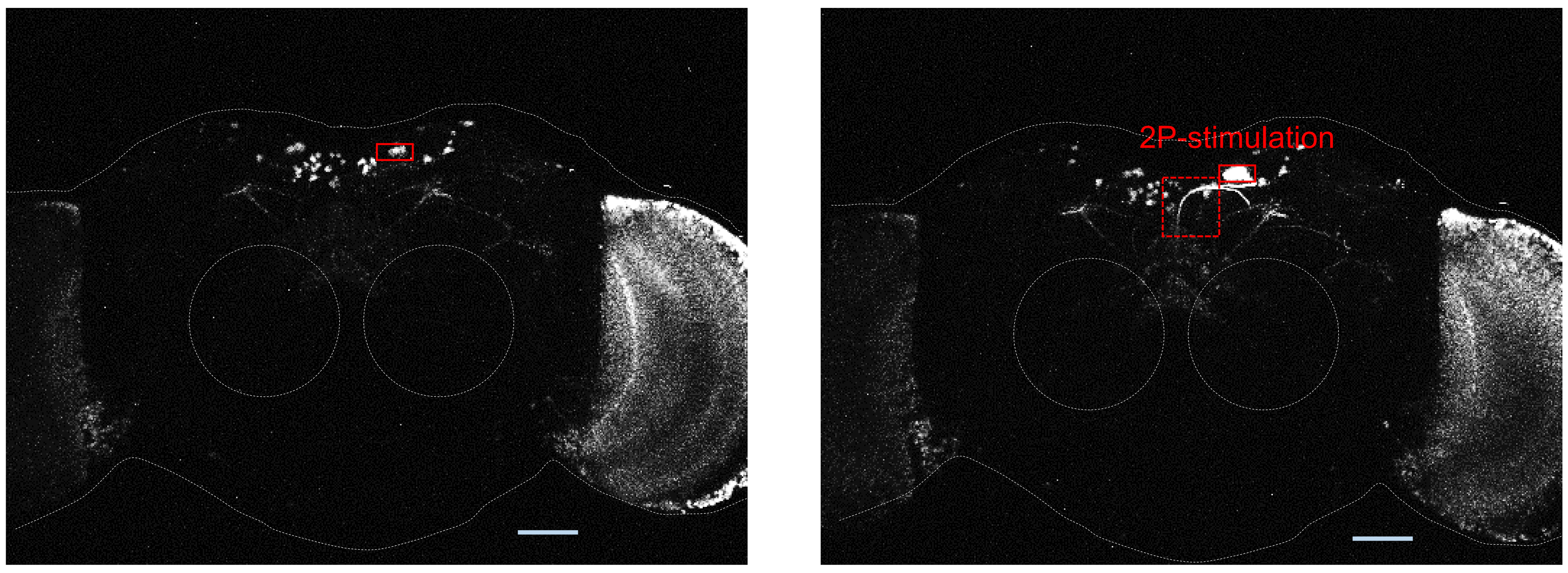

Pre-stimulation

Post-stimulation

Fig. S5 SMP DTK^{+} neurons send their neurites to the PI region. After the stimulation of the cell bodies of SMP DTK^{+} neurons expressing PA-GFP (left), their neurites could be visualized extending to the PI region (right). Scale bars, 10 μm.
