# Supplementary material for: A novel satiety sensor detects circulating glucose and suppresses food consumption via insulin-producing cells in Drosophila
Source: Cell Res. 2020 Dec 3;31(5):580–8. doi: 10.1038/s41422-020-00449-7 (PMC8089096; doi:10.1038/s41422-020-00449-7)
Supplement: Supplementary file 6 — Supplementary information, Figure S6 [file 41422_2020_449_MOESM6_ESM.pdf]

Fig S6

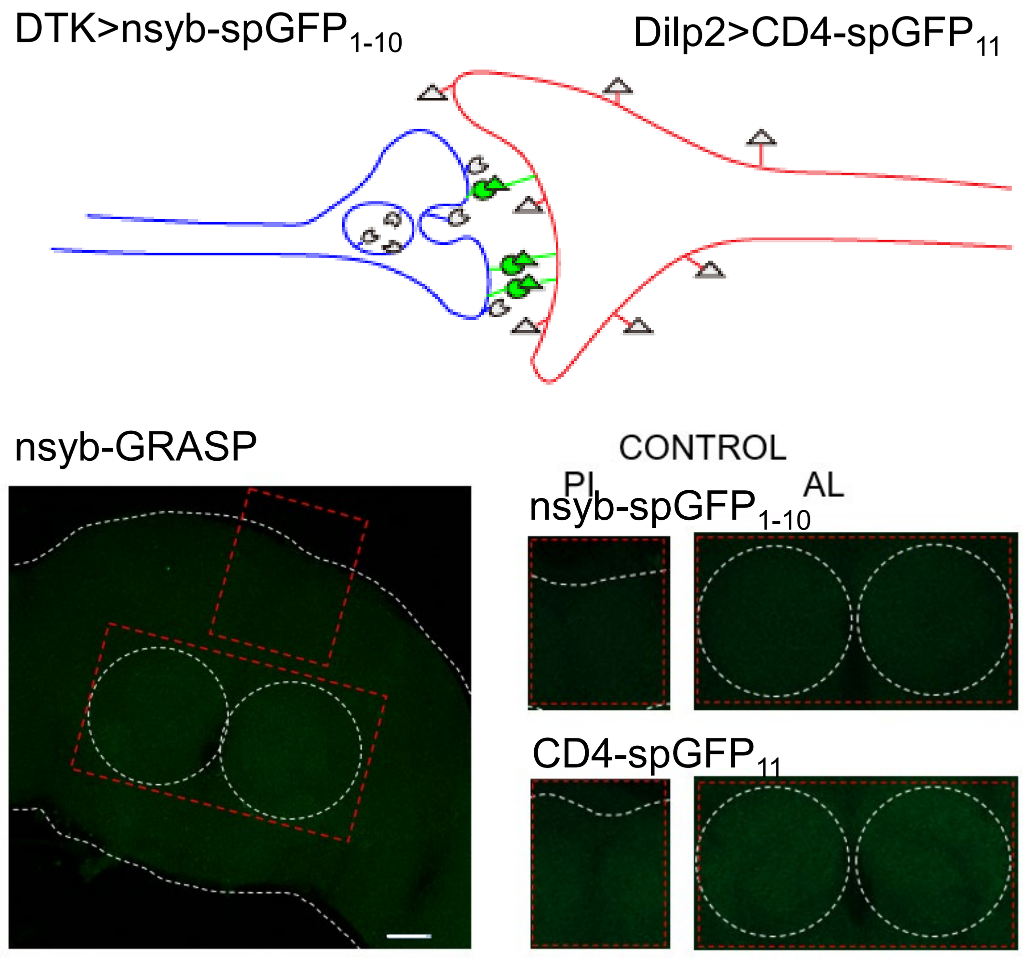

DTK>nsyb-spGFP1-10
Dilp2>CD4-spGFP11
nsyb-GRASP
CONTROL
PI
AL
nsyb-spGFP1-10
CD4-spGFP11

Fig. S6 DTK$^+$ neurons and IPCs do not have direct synaptic connections. Schematic diagram of nSyb-GRASP between DTK$^+$ and IPCs (top). The absence of nSyb-GRASP signals between DTK$^+$ neurons and IPCs in the PI region and the AL region (red boxes) (left). In the control groups lacking either of the nSyb-GRASP component, no nSyb-GRASP signal was seen (right). Scale bar, 10 μm.
