# Supplementary material for: A novel satiety sensor detects circulating glucose and suppresses food consumption via insulin-producing cells in Drosophila
Source: Cell Res. 2020 Dec 3;31(5):580–8. doi: 10.1038/s41422-020-00449-7 (PMC8089096; doi:10.1038/s41422-020-00449-7)
Supplement: Supplementary file 7 — Supplementary information, Figure S7 [file 41422_2020_449_MOESM7_ESM.pdf]

Fig S7

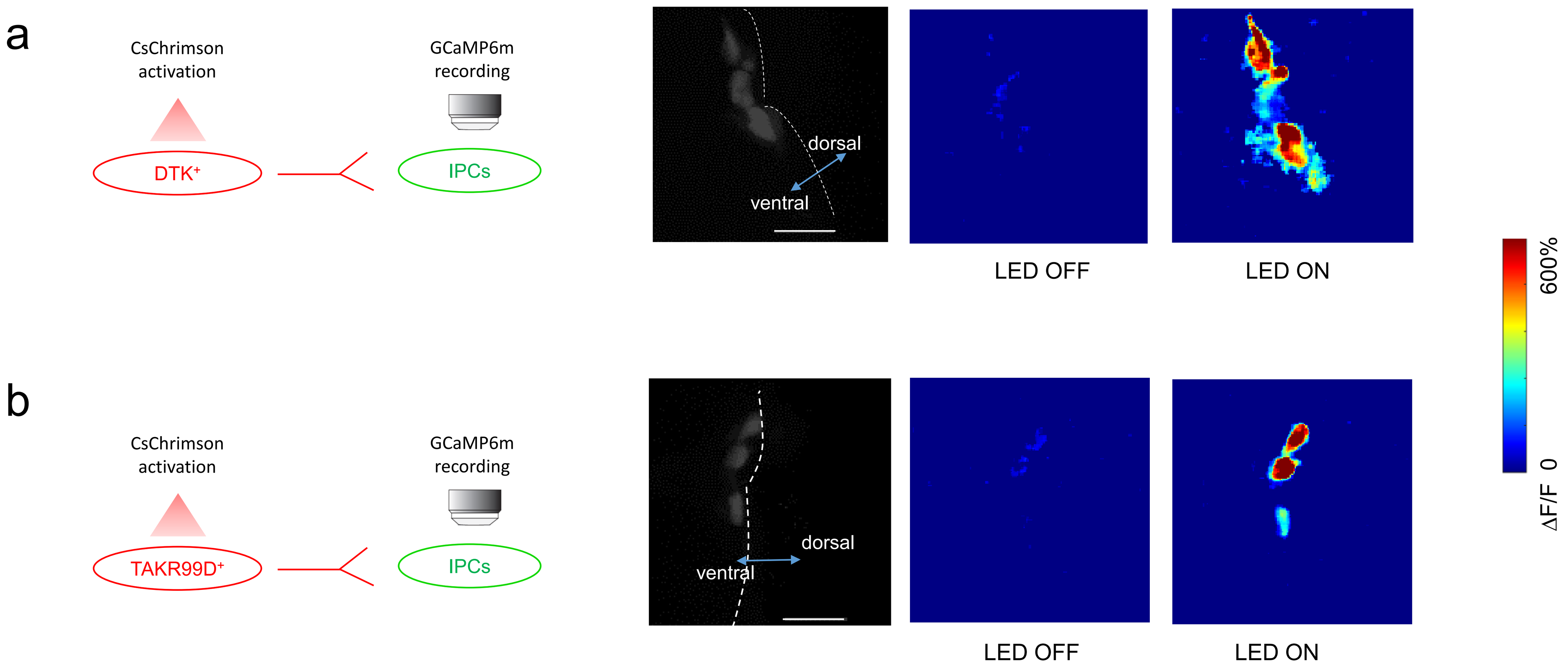
a
CsChrimson activation
GCaMP6m recording
DTK⁺
IPCs
dorsal
ventral
LED OFF
LED ON
600%
ΔF/F 0
b
CsChrimson activation
GCaMP6m recording
TAKR99D⁺
IPCs
dorsal
ventral
LED OFF
LED ON

Fig. S7 IPCs can be activated by the activation of DTK$^+$ and TAKR99D$^+$ neurons.

a In the *ex vivo* calcium imaging preparations, the opto-activation of DTK$^+$ neurons elicited robust calcium responses in IPCs. **b** In the *ex vivo* calcium imaging preparations, the opto-activation of TAKR99D$^+$ neurons elicited robust calcium responses in IPCs.
