# Supplementary material for: A novel satiety sensor detects circulating glucose and suppresses food consumption via insulin-producing cells in Drosophila
Source: Cell Res. 2020 Dec 3;31(5):580–8. doi: 10.1038/s41422-020-00449-7 (PMC8089096; doi:10.1038/s41422-020-00449-7)
Supplement: Supplementary file 8 — Supplementary information, Figure S8 [file 41422_2020_449_MOESM8_ESM.pdf]

Fig S8

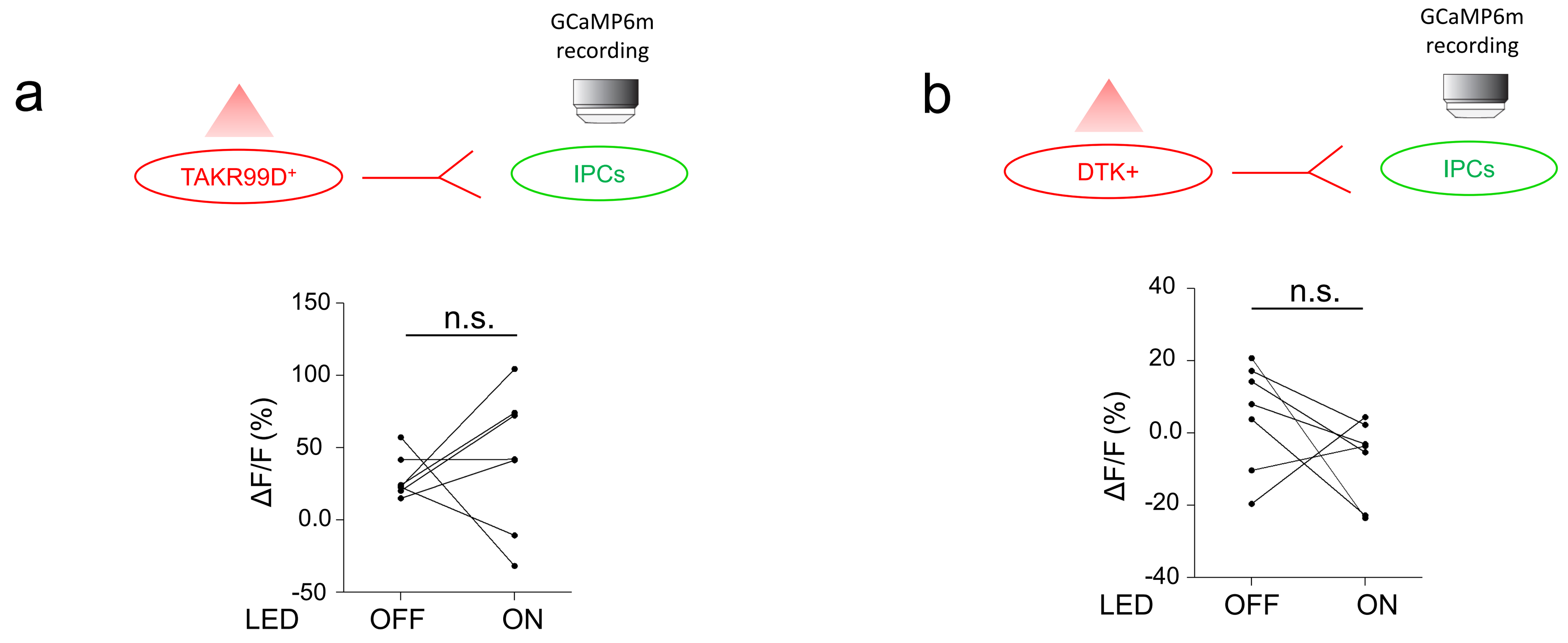
a
GCaMP6m recording
TAKR99D+
IPCs
150
100
50
0.0
-50
n.s.
ΔF/F (%)
LED
OFF
ON
b
GCaMP6m recording
DTK+
IPCs
40
20
0.0
-20
-40
n.s.
ΔF/F (%)
LED
OFF
ON

Fig. S8 Without CsChrimson, red light stimulation does not elicit calcium responses in IPCs. Quantification of the calcium responses of IPCs upon the red light stimulation without CsChrimson expression in TAKR99D^{+} (**a**) and DTK^{+} (**b**) neurons (n = 6–7). ns, $P > 0.05$.
