# Supplementary material for: A novel satiety sensor detects circulating glucose and suppresses food consumption via insulin-producing cells in Drosophila
Source: Cell Res. 2020 Dec 3;31(5):580–8. doi: 10.1038/s41422-020-00449-7 (PMC8089096; doi:10.1038/s41422-020-00449-7)
Supplement: Supplementary file 9 — Supplementary information, Figure S9 [file 41422_2020_449_MOESM9_ESM.pdf]

Fig S9

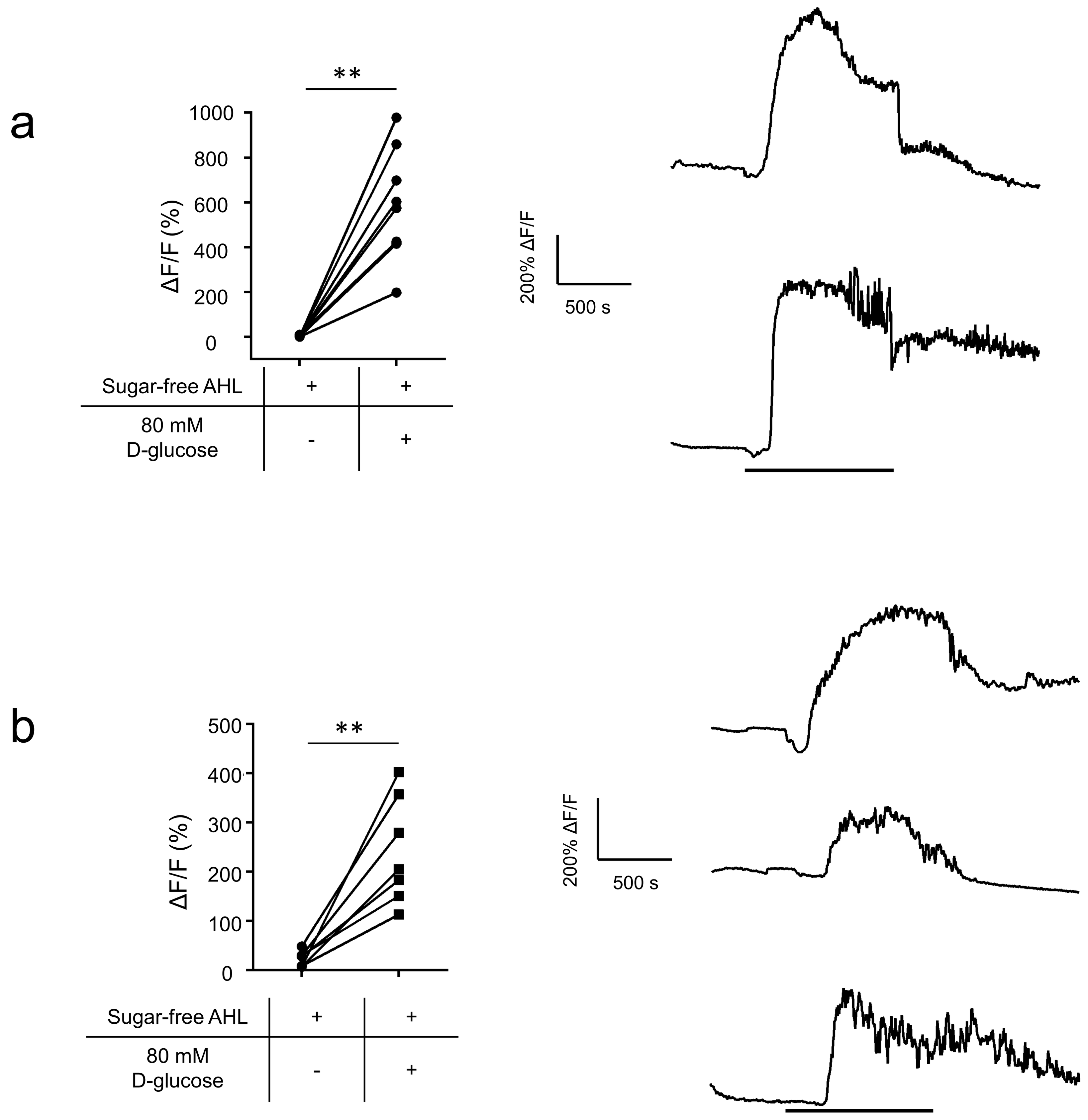
a
**
1000
800
600
400
200
0
ΔF/F (%)
Sugar-free AHL
80 mM
D-glucose
+
+
-
+
200% ΔF/F
500 s
b
**
500
400
300
200
100
0
ΔF/F (%)
Sugar-free AHL
80 mM
D-glucose
+
+
-
+
200% ΔF/F
500 s

Fig. S9 Calcium responses in TAKR99D$^+$ neurons and IPCs upon glucose perfusion. **a** Quantification (left) and representative traces (right) of the calcium responses of TAKR99D$^+$ neurons to 80 mM D-glucose in the *ex vivo* calcium imaging preparations (n = 7). **b** The calcium responses of IPCs to 80 mM D-glucose in the *ex vivo* calcium imaging preparations (n = 7). $^{**}P < 0.01$.
