# Supplementary material for: A novel satiety sensor detects circulating glucose and suppresses food consumption via insulin-producing cells in Drosophila
Source: Cell Res. 2020 Dec 3;31(5):580–8. doi: 10.1038/s41422-020-00449-7 (PMC8089096; doi:10.1038/s41422-020-00449-7)
Supplement: Supplementary file 10 — Supplementary information, Figure S10 [file 41422_2020_449_MOESM10_ESM.pdf]

Fig S10

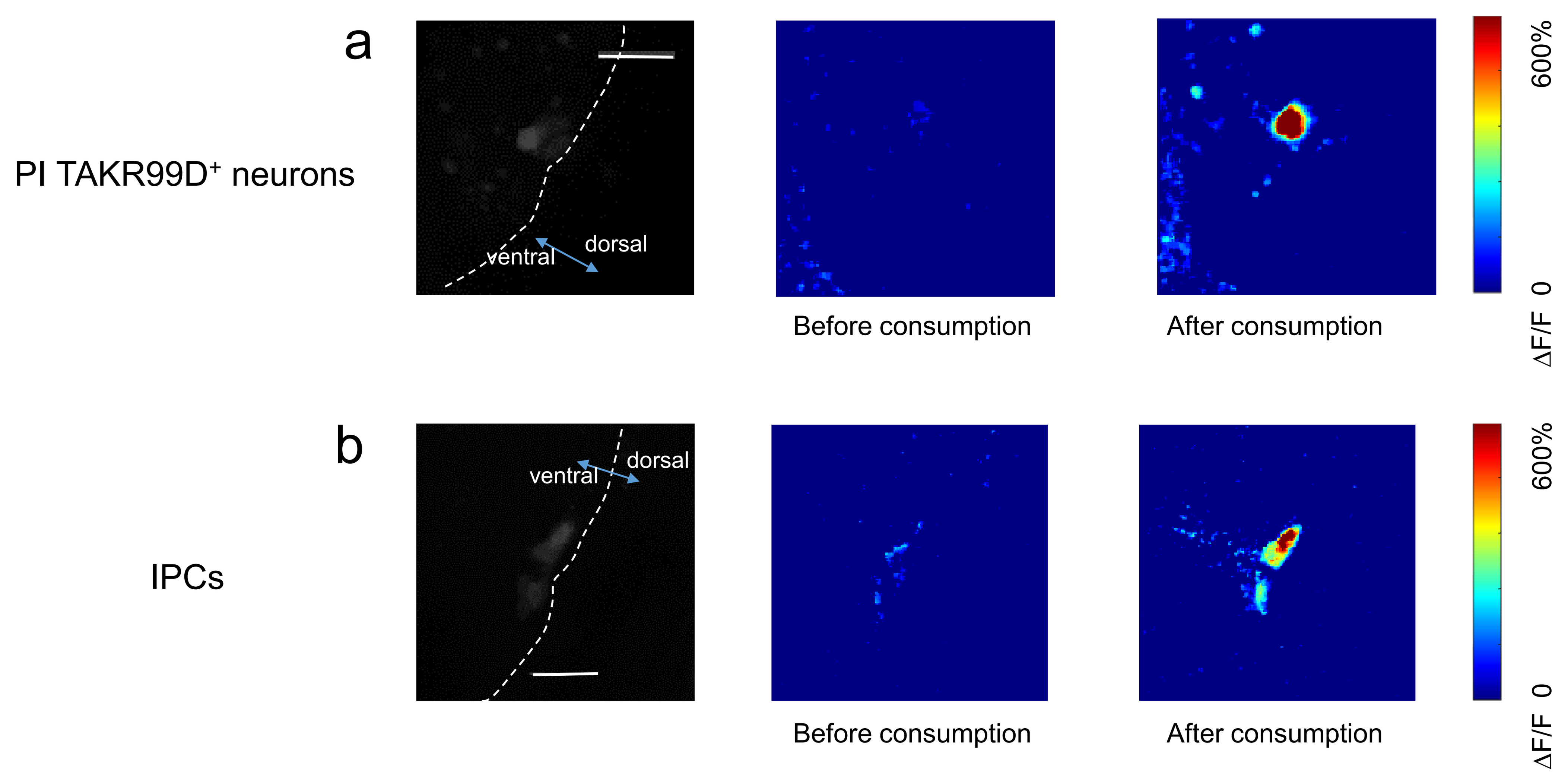
a
PI TAKR99D+ neurons
ventral
dorsal
Before consumption
After consumption
600%
0
ΔF/F
b
IPCs
ventral
dorsal
Before consumption
After consumption
600%
0
ΔF/F

Fig. S10 Calcium responses in TAKR99D$^+$ neurons and IPCs upon food ingestion.

a The calcium responses of PI TAKR99D$^+$ neurons to the ingestion of D-glucose in the *in vivo* calcium imaging preparations (n = 10). **b** The calcium responses of IPCs to the ingestion of D-glucose in the *in vivo* calcium imaging preparations (n = 12).
