# Supplementary material for: A novel satiety sensor detects circulating glucose and suppresses food consumption via insulin-producing cells in Drosophila
Source: Cell Res. 2020 Dec 3;31(5):580–8. doi: 10.1038/s41422-020-00449-7 (PMC8089096; doi:10.1038/s41422-020-00449-7)
Supplement: Supplementary file 11 — Supplementary information, Figure S11 [file 41422_2020_449_MOESM11_ESM.pdf]

Fig S11

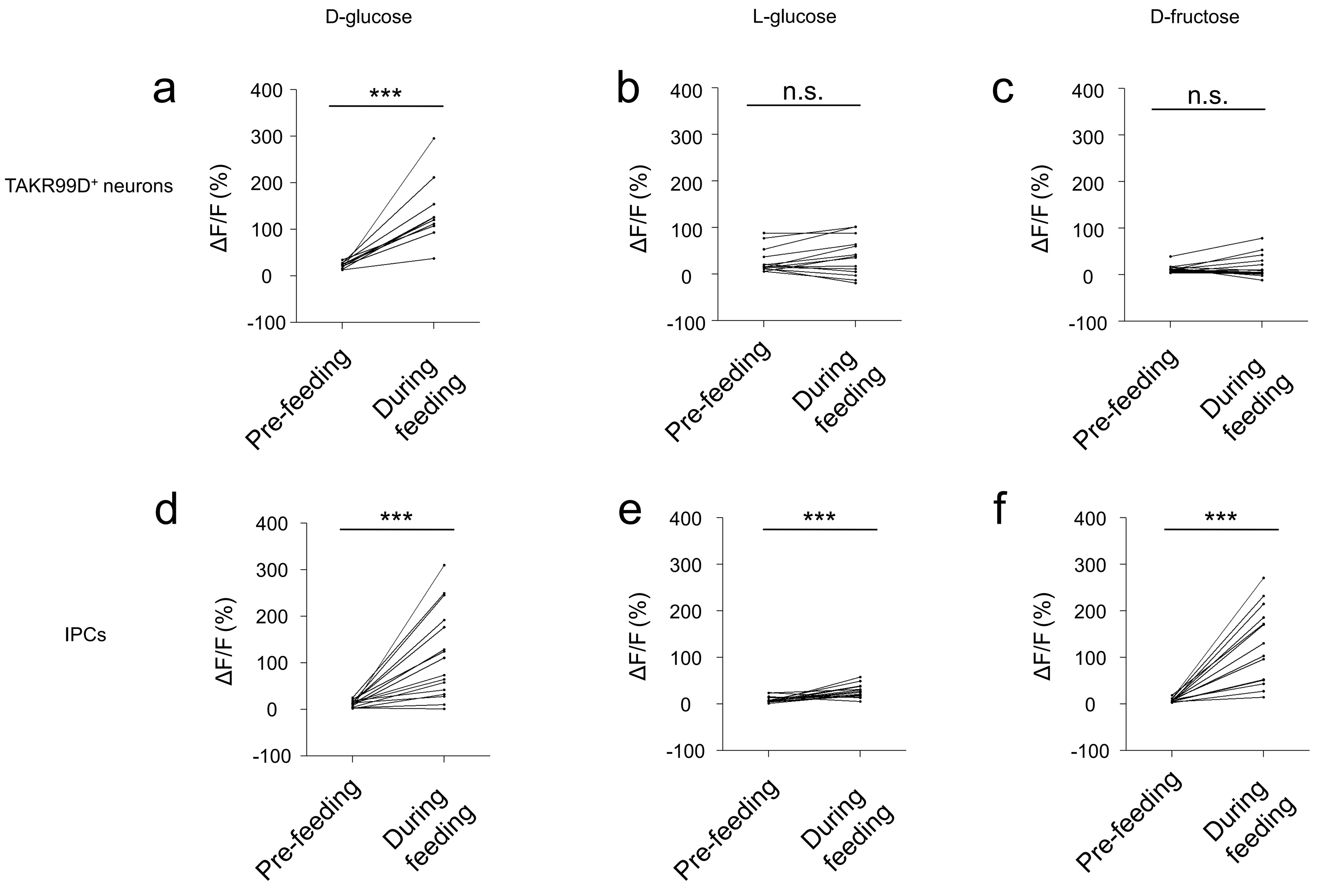
D-glucose
L-glucose
D-fructose
TAKR99D^{+} neurons
IPCs
a
b
c
d
e
f
***
n.s.
ΔF/F (%)
400
300
200
100
0
-100
Pre-feeding
During feeding

Fig. S11 Quantification of calcium responses in TAKR99D$^+$ neurons and IPCs upon food ingestion. a-c Quantification of the calcium responses of PI TAKR99D$^+$ neurons upon the ingestion of D-glucose **(a)**, L-glucose **(b)**, and D-fructose **(c)** (n = 10–16). **d-f** Quantification of the calcium responses of IPCs upon the ingestion of D-glucose **(d)**, L-glucose **(e)**, and D-fructose **(f)** (n = 15–16).
